# Differential response to sulfur nutrition of two common bean genotypes differing in storage protein composition

**DOI:** 10.3389/fpls.2015.00092

**Published:** 2015-02-20

**Authors:** Sudhakar Pandurangan, Mark Sandercock, Ronald Beyaert, Kenneth L. Conn, Anfu Hou, Frédéric Marsolais

**Affiliations:** ^1^Department of Biology, University of Western OntarioLondon, ON, Canada; ^2^Genomics and Biotechnology, Southern Crop Protection and Food Research Centre, Agriculture and Agri-Food Canada, London, ON, Canada; ^3^Cereal Research Centre Morden, Agriculture and Agri-Food CanadaCanada, Morden, MB, Canada

**Keywords:** sulfur nutrition, seed yield, sulfur amino acids, globulin, lipoxygenase, albumin, lectin, common bean

## Abstract

It has been hypothesized that the relatively low concentration of sulfur amino acids in legume seeds might be an ecological adaptation to nutrient poor, marginal soils. SARC1 and SMARC1N-PN1 are genetically related lines of common bean (dry bean, *Phaseolus vulgaris*) differing in seed storage protein composition. In SMARC1N-PN1, the lack of phaseolin and major lectins is compensated by increased levels of sulfur-rich proteins, resulting in an enhanced concentration of cysteine and methionine, mostly at the expense of the abundant non-protein amino acid, *S*-methylcysteine. To identify potential effects associated with an increased concentration of sulfur amino acids in the protein pool, the response of the two genotypes to low and high sulfur nutrition was evaluated under controlled conditions. Seed yield was increased by the high sulfate treatment in SMARC1N-PN1. The seed concentrations of sulfur, sulfate, and *S*-methylcysteine were altered by the sulfur treatment in both genotypes. The concentration of total cysteine and extractible globulins was increased specifically in SMARC1N-PN1. Proteomic analysis identified arcelin-like protein 4, lipoxygenase-3, albumin-2, and alpha amylase inhibitor beta chain as having increased levels under high sulfur conditions. Lipoxygenase-3 accumulation was sensitive to sulfur nutrition only in SMARC1N-PN1. Under field conditions, both SARC1 and SMARC1N-PN1 exhibited a slight increase in yield in response to sulfur treatment, typical for common bean.

## INTRODUCTION

Common bean (dry bean, *Phaseolus vulgaris*) is an important source of protein and fiber in human diets. Like other grain legumes, its protein quality is sub-optimal, being limited by the levels of the essential sulfur amino acids, methionine, and cysteine. During the past decades, a lot of effort has been dedicated to improving protein quality in grain legumes, primarily using transgenic approaches (Krishnan, 2005; Ufaz and Galili, 2008; Amir et al., 2012; Galili and Amir, 2013). Transgenic expression of foreign proteins can be limited by the supply of sulfur, and often results in a shift of sulfur away from endogenous, sulfur-rich proteins (Streit et al., 2001; Tabe and Droux, 2002; Chiaiese et al., 2004). **Table 1** lists the different experiments that were performed involving transgenic expression of sulfur-rich proteins in legumes and their outcomes. In *Vicia narbonensis*, co-expression of Brazil nut 2S albumin and a feedback-insensitive, bacterial aspartate kinase was associated with increased sulfur concentration in seed (Demidov et al., 2003). A common concern with these approaches is the potential allergenicity of the foreign proteins (Nordlee et al., 1996; Kelly and Hefle, 2000; Krishnan et al., 2010). A possible solution to this problem is the expression of a *de novo* synthetic protein, MB-16. An alternative approach involves the transgenic manipulation of sulfur amino acid pathways. Overexpression of cytosolic serine acetyltransferase in developing soybean seed led to a 70% increase in total cysteine concentration (Kim et al., 2012). Expression of a feedback-insensitive *Arabidopsis* cystathionine γ-synthase (AtD-CGS), encoding a protein lacking 30 amino acids in the N-terminal domain, raised total methionine concentration by 1.8 to 2.3-fold, with an overall increase in seed protein concentration (Song et al., 2013). By contrast, expression of the feedback-insensitive *mto1-1* allele, harboring a point mutation, led to elevated levels of free methionine, but not total methionine in soybean, whereas in azuki bean, the levels of cystathionine were raised while total methionine concentration was actually decreased (Hanafy et al., 2013a,b). A completely different approach proposed to improve protein quality in common bean involves the introduction of highly digestible phaseolin types from wild accessions by conventional breeding (Montoya et al., 2010). Based on *in vitro* protein digestibility corrected amino acid score, genotypes having highly digestible phaseolin types could increase bioavailable sulfur amino acids by approximately 30% as compared with S type phaseolin present in Mesoamerican cultivars.

**Table 1:** Attempts to improve sulfur amino acid concentration in legumes by transgenic expression of sulfur-rich proteins.

Crop plant	Foreign protein	Increase in sulfur amino acids	Reference
Soybean	Brazil nut 2S albumin	Methionine 26%	Townsend and Thomas (1994)
Soybean	15 kDa δ-zein	Methionine by 20% and cysteine by 35%	Dinkins et al. (2001)
Soybean	27 kDa γ-zein	Methionine by 19% and cysteine by 30%	Li et al. (2005)
Soybean	11 kDa δ-zein	Methionine (alcohol soluble fraction)	Kim and Krishnan (2004)
Common bean	Brazil nut 2S albumin	Methionine by 20%	Arag ao et al. (1999)
Lupin	Sunflower seed albumin	Methionine by 90%	Molvig et al. (1997), Tabe and Droux (2002)
Chickpea	Sunflower seed albumin	Methionine by 90%	Chiaiese et al. (2004)
*Vicia narbonensis*	Brazil nut 2S albumin and feedback-insensitive aspartate kinase	Methionine by 100% and cysteine by 20%	Demidov et al. (2003)
Soybean	MB-16	Methionine by 16% and cysteine by 66%	Zhang et al. (2014)

Grain yield in legumes has a low heritability due to environmental variables. Consequently, agronomic practices combined with proper fertilizer management heavily influence yield. Sulfur, which has long been known to play a major role in plant metabolism (Takahashi et al., 2011), increases yield in common bean (Malavolta et al., 1987) and influences seed quality via the proportion of sulfur containing amino acids, cysteine, and methionine. Sulfate is the most significant and readily mobilized form of sulfur. Sulfate taken up by the roots is reduced to sulfide and further incorporated into cysteine. Cysteine is converted to methionine or incorporated into glutathione and proteins. Sulfate, and/or organic forms of sulfur, such as glutathione (Anderson and Fitzgerald, 2001) or *S*-methylmethionine (Bourgis et al., 1999; Lee et al., 2008; Tan et al., 2010), is transported through the phloem, followed by uptake by transporters into the developing embryo and translocation between seed tissues (Zuber et al., 2010). Delivery of adequate sulfur to seed tissues is needed for maximizing production and to improve protein quality (Hawkesford and De Kok, 2006). Nutrient status of the plant regulates the uptake and assimilation of sulfate (Smith et al., 1995; Buchner et al., 2004). Studies have shown that a decrease in sulfate availability results in a several-fold enhanced expression of sulfate transporter genes, which enhances the capacity for sulfate uptake (Hawkesford, 2000, 2003). Sulfur fertilization favorably affects protein quality by increasing the expression of proteins rich in sulfur amino acids. Control of seed protein accumulation by the sulfur status has been well documented in several legumes, including globulins in soybean and lupine (Blagrove et al., 1976; Gayler and Sykes, 1985), and globulins, and albumins in pea (Chandler et al., 1983, 1984; Higgins et al., 1987). Reduced expression of pea legumin and albumin 1 genes in response to sulfur deficiency was further confirmed in transgenic tobacco (Rerie et al., 1991; Morton et al., 1998). In general, high sulfur stimulates the expression of sulfur-rich globulins and albumins while sulfur deficiency increases the expression of sulfur-poor globulins. In soybean, the accumulation of the sulfur-poor β-subunit of β-conglycinin is repressed by exogenous methionine (Holowach et al., 1984, 1986). This was confirmed in transgenic *Arabidopsis* (Naito et al., 1995). The immediate metabolic precursor of cysteine, *O*-acetylserine, seems involved in the up-regulation of the β-subunit of β-conglycinin under conditions of sulfur deficiency (Kim et al., 1999). A high nitrogen to sulfur ratio not only increases the accumulation of the β-subunit of β-conglycinin, but also reduces the levels of sulfur-rich Bowman-Birk inhibitor (Krishnan et al., 2005). Recent research has focused on adaptation of legumes to sulfur deficiency, highlighting the possible role of a vacuolar sulfate transporter in *Medicago truncatula* (Zuber et al., 2013). This research is relevant to improvement of sulfur use efficiency (De Kok et al., 2011).

Crop plants mitigate the effect of silencing or deficiency in storage proteins through rebalancing of the seed proteome (Marsolais et al., 2010; Herman, 2014; Wu and Messing, 2014). SARC1 and SMARC1N-PN1 are related genotypes of common bean differing in seed protein composition (Osborn et al., 2003). They share 87.5 and 83.6% of the recurrent, Sanilac parental background, respectively. SARC1 integrates the lectin arcelin-1 from a wild accession. SMARC1N-PN1 lacks phaseolin and major lectins, through introgressions from a *P. coccineus* accession and Great Northern 1140, respectively. These changes are associated with an increased concentration of methionine and cysteine, by 10 and 70%, respectively, concomitant with 70% decrease in *S*-methylcysteine concentration (Taylor et al., 2008). Proteomic and transcript profiling indicated that several sulfur-rich proteins have increased levels in SMARC1N-PN1, including the 11S globulin legumin, albumin-2, defensin D1, Bowman-Birk type proteinase inhibitor 2, albumin-1, basic 7S globulin, and Kunitz trypsin protease inhibitor (Marsolais et al., 2010; Yin et al., 2011; Liao et al., 2012). SARC1 and SMARC1N-PN1 offer a unique system to investigate how related legume genotypes, harboring natural genetic variation in storage protein composition, respond to sulfur deficiency. The presence of an endogenous sink for sulfur in SMARC1N-PN1 is associated with an increased plasticity of the seed composition in response to sulfur nutrition.

## MATERIALS AND METHODS

### PLANT MATERIALS AND GROWTH CONDITIONS

SARC1 and SMARC1N-PN1 were evaluated for their response to sulfur nutrition by fertilizing with a nutrient solution containing low sulfur (LS) or high sulfur (HS) as described in previous work with common bean (Sánchez et al., 2002) and chickpea (Chiaiese et al., 2004), with modifications (Pandurangan et al., submitted to *Sulfur Metabolism in Plants. Molecular Physiology and Ecophysiology of Sulfur. Proceedings of the International Plant Sulfur Workshop*). Seeds were sown in small trays containing vermiculite for better germination. Ten day old seedlings were transplanted to pots (17 cm× 20 cm) containing sand, perlite, and vermiculite in a 2:1:1 ratio. The experimental unit consisted in a pot with two plants. There were two groups (LS and HS) of five pots for each genotype. For the initial establishment, the transplanted seedlings were fertilized once with 20:20:20 (N:P:K; Plant Prod, Brampton, ON, Canada) before the actual sulfur treatment. The nutrient solutions for the treatment were made fresh from stock solutions and applied once weekly. The LS nutrient solution contained 0.2 mM K_2_SO_4_ and 1.8 mM MgCl_2_; HS contained 0.2 mM K_2_SO_4_ and 1.8 mM MgSO_4_. Other nutrients included 4.5 mM Ca(NO_3_)_2_, 1.7 mM K_2_HPO_4_, 4 μM MnSO_4_.H_2_O, 5 μM H_3_BO_3_, 10 μM Fe-EDTA, 0.25 μM CuSO_4_.5H_2_O, 1 μM ZnSO_4_.7H_2_O, and 0.2 μM Na_2_MoO_4_.2H_2_O. Plants were grown in cabinets (Conviron E8H, Winnipeg, MB, Canada) with 16 h light (300–400 μmol photons m^-2^ s^-1^) and 8 h dark, with a temperature cycling between 18 and 24°C (Pandurangan et al., 2012).

### FIELD TRIAL

The response of SARC1 and SMARC1N-PN1 genotypes to sulfur fertilization was assessed in a field trial conducted at the Cereal Research Centre Morden, MB, Canada, in 2012. Soil was sampled in the fall 2011 and analyzed at Exova, Calgary, AB, Canada, to determine the amount of nutrients needed for the treatment. Nutrient analysis found 24:129:1345 kg ha^-1^ as nitrogen, phosphorus, potassium, and 47 kg ha^-1^ as sulfur. Crops were either grown with or without applied sulfur (30 kg ha^-1^) as gypsum (CaSO_4_⋅2H_2_O). Recommended seed rate (250,000 seeds ha^-1^ = 25 seed m^-2^) and cultural practices were used at all plots. Plot size was 1 m × 5.5 m trimmed to 5.0 m^2^ with spacing of two rows at 0.5 m between plots. All plots were planted in a randomized complete block design with four replications for each treatment, each replication consisting of two rows of 5.5 m long accounting for 550 seeds per treatment. Two adjacent rows represented one replicate. A post emergent herbicide, Basagran (BASF Canada, Mississauga, ON, Canada) was applied at the rate of 2.2 l ha^-1^. Fertilizer added in all the treatment plots was 120 kg N ha^-1^. Dry mature seeds from the net area of each plot were harvested separately, weighed, and recorded as seed yield (kg ha^-1^).

### AMINO ACID ANALYSIS

Extraction and quantification of sulfur amino acids from mature seed tissue was performed as previously described, using HPLC after derivatization with phenylisothiocyanate (Hernández-Sebastià et al., 2005; Taylor et al., 2008). Cysteine was quantified separately as cysteic acid after oxidation with performic acid.

### ALBUMIN AND GLOBULIN EXTRACTION AND QUANTIFICATION

Albumin and globulin fractions were extracted from mature seed as described by Rolletschek et al. (2005). Protein in the extracts was quantified using the Bio-Rad Protein Assay reagent (Mississauga, ON, Canada) with bovine serum albumin as standard. Protein concentration was normalized according to the volume of extract recovered. A volume of sample equivalent to the same weight of tissue extracted was subjected to SDS-PAGE on a 12% polyacrylamide gel. Following staining with Coomassie R-250, band intensities in globulin extracts were measured with Quantity One 4.2.1 (Bio-Rad). Quantity One is very tolerant of an assortment of electrophoretic artifacts, and can measure total and average quantities, determine relative and actual amounts of protein. Prior to quantification the image acquired from scanning the gels was optimized by the software by performing lane background subtraction to reduce any noise or background density while maintaining image quality followed by filtering to remove small noise features while leaving larger features relatively unaffected. The software was used for identifying lanes and defining, quantitating, and matching bands. Lane-based quantitation used to calculate intensity of similar bands across lanes involves calculating the average intensity of pixels across the band width and integrating over the band height. The quantity of a band as measured by the area under its intensity profile curve is expressed as intensity × mm. Apparent molecular mass was calculated based on standards using the same software.

### SAMPLE PREPARATION AND MASS SPECTROMETRY

Proteomic experiments were performed at the London Regional Proteomics Centre of the University of Western Ontario. Sample preparation was carried out at the Functional Proteomics Facility. Protein bands of interest identified by band intensities in the globulin extracts were excised by the robotic Ettan Spot Picker (GE Healthcare Life Sciences, Baie d’Urfé, QC, Canada) and suspended in 50% methanol and 5% acetic acid for digestion. Trypsin digestion was performed using the MassPREP automated digester (Waters, Mississauga, ON, Canada). Gel pieces were destained using 50 mM ammonium bicarbonate and 50% acetonitrile followed by protein reduction with 10 mM dithiothreitol, alkylation with 55 mM iodoacetamide and tryptic digestion. Peptides were extracted using a solution of 1% formic acid and 2% acetonitrile and lyophilized. Peptides were dissolved in a solution of 30% acetonitrile and 0.1% trifluoroacetic acid mixed with α-cyano-4-hydroxycinnamic acid in 50% acetonitrile, 12.5 mM ammonium citrate, 0.1% trifluoroacetic acid, and analyzed on a 4700 Proteomics Discovery System (Life Technologies, Burlington, ON, Canada) at the MALDI-MS facility. MS analysis was carried out in an *m/z* range of 500–3500 and mass tolerance of 50 ppm. Data acquisition and processing were done using 4000 Series Explorer and Data Explorer (Life Technologies). The instrument was equipped with a 355 nm Nd:YAG laser and the laser rate was 200 Hz. Reflectron and linear positive ion modes were used. Each mass spectrum was collected as a sum of 1000 shots. Samples from protein bands no. 1, 4, and 5 were further analyzed by LC–MS–MS at the Biological Mass Spectrometry Laboratory. They were reconstituted in 18 μl of 0.1% formic acid in water and 10 μl was injected into the UPLC-MS/MS system. The system was comprised of a Waters nanoAcquity UPLC with a Waters C18 trapping and Waters 25 cm analytical column coupled to a Waters QToF Ultima Global Mass Spectrometer. The sample was run at a flow rate of 0.3 μl/min. Solvent A was water:formic acid 0.1% and solvent B was acetonitrile:formic acid 0.1%. Solvent B was set to go from 5% to 60% in 40 min and then reach 95% by 42.5 min. B was kept at 95% for 5 min and brought back to 5% at 50 min. The column was re-equilibrated for 25 min prior to the following injection. Sample loading took 3 min with a flow rate of 10 μl/min at 99% A and 1% B. MS survey scan was performed at a cone voltage of 35 V and set to 1.4 s with 0.1 s interscan and recorded from 300 to 1800 *m/z*. In a given survey scan, all doubly and triply charged ions with intensities greater than 40 counts were considered candidate to undergo MS/MS fragmentation. MS/MS acquisition would stop as soon as the total ion current would reach 25000 counts per second or after a maximum time of 6 s. MS/MS scan was acquired from 50 to 1800 *m/z* for 1.4 s with an interscan time of 0.1 s. Selected ions were fragmented with a collision energy of 30 eV.

Peptide mass fingerprint data were analyzed by searching peptide mass values against a translated version of the preliminary release of the common bean genome (June 26, 2012; Schmutz et al., 2014) using MASCOT (Matrix Science, Boston, MA, USA). The following parameters were used: 1 missed cleavage; fixed carbamidomethyl alkylation of cysteine; variable oxidation of methionine; peptide mass tolerance: ± 1.2 Da; peptide charge state: +1, significant threshold: *p* < 0.05. For MS-MS, raw data were converted to mgf files using PEAKS 5.3 (Bioinformatics Solutions Inc., Waterloo, ON, Canada). MS/MS ion search was performed with MASCOT against the same database, as well the Mascot database (MSDB, August 31, 2006) using similar cleavage and post-translational modification parameters.

### SULFATE ANALYSIS

Replicate samples (∼50 seeds) were ground to a fine powder in a Kleco Ball Mill (Visalia, CA, USA) and lyophilized. Approximately 100 mg of ground tissue was used for sulfate analysis by chemical suppression ion chromatography and conductivity detection using a Dionex DX-600 Ion Chromatograph (Thermo Fisher Scientific, Sunnyvale, CA, USA), as described in Herschbach et al. (2000) with modifications. Approximately 100 mg of tissue was extracted in 0.5 ml of deionized water. The suspension was centrifuged at 16,000 ×*g* for 10 min at 4°C. A 300 μl aliquot of the cleared supernatant was transferred to an ion chromatography vial for testing using an IonPac anion-exchange column (AS14A, 4 mm; Thermo Fisher Scientific) and eluted with a mixture of 3.5 mM sodium hydrogen carbonate, and 1.0 mM sodium carbonate. A 10 μl aliquot of the solution contained in vials was injected into the eluent stream and background conductivity of eluents reduced by a suppressor (Anion Self-Regenerating Suppressor Ultra, 4 mm). An AS50 auto sampler equipped with a refrigerated chamber was used to house the vials and Dionex Peaknet 6.0 software was employed to track and analyze data.

### ELEMENTAL ANALYSIS

Approximately 500 mg of ground seed tissue was submitted to elemental analysis which was performed by dry combustion with a CNS-2000 Elemental Analyzer (LECO Instruments ULC, Mississauga, ON, Canada) as described by Taylor et al. (2008).

### STATISTICAL ANALYSIS

Analysis of variance was performed using SAS version 9.2 (Toronto, ON, Canada). Homogeneity of the variances was inspected by residual graphic analysis.

### ACCESSION NUMBERS

Accession numbers for proteins in this study are as follows: arcelin-like 4 [Uniprot:Q8RVX7]; lipoxygenase-3 [Phytozome:Phvul.005g157000.1]; albumin-2 [Phytozome:Phvul007g275800]; α-amylase inhibitor β-subunit [Uniprot:Q9S9E1]; α-amylase inhibitor 1 [Uniprot:Q6J2U4].

## RESULTS

### INCREASED YIELD OF SMARC1N-PN1 IN RESPONSE TO HIGH SULFUR UNDER CONTROLLED CONDITIONS

**FIGURE 1:**
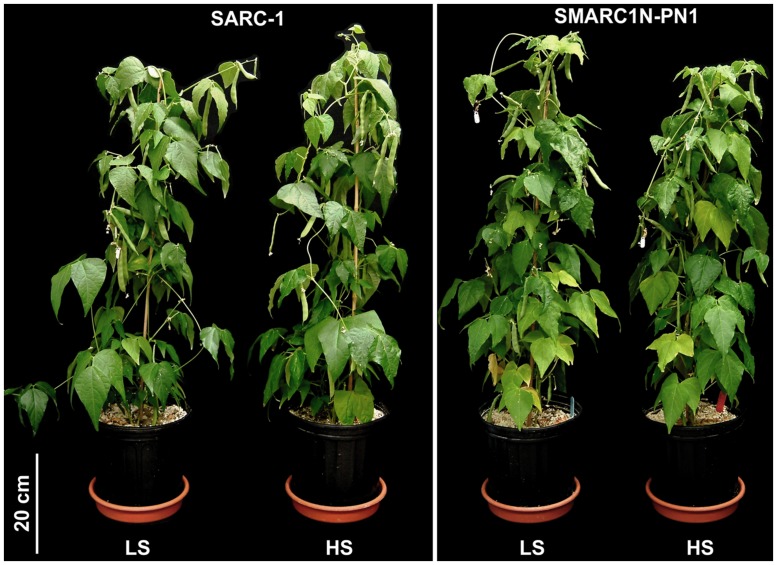
**Plants grown under controlled conditions with low sulfur (LS) and high sulfur (HS), 60 days after germination and 26 days after flowering.** Vegetative growth appeared similar between genotypes.

To determine whether differences in response to sulfur nutrition are associated with the presence of an additional, endogenous sink for sulfur in SMARC1N-PN1, an experiment was performed under controlled conditions with two levels of sulfate fertilization. Treatment conditions were designed so that the LS conditions correspond to a sulfur deficiency at the reproductive stage. The LS condition was found to be non-limiting for vegetative growth (**Figure 1**). The nitrogen levels selected are non-limiting (Sánchez et al., 2002; Chiaiese et al., 2004). The two genotypes were compared for their agronomic parameters. The following variables were evaluated: number of seeds, seed weight, and seed yield (**Table 2**). The fact that SARC1 and SMARC1N-PN1 are not completely isogenic explains the occurrence of genotypic differences for some of these characteristics. There were significant interactions between factors for seed weight and yield. Whereas the average seed weight decreased under HS for SARC1, it actually increased for SMARC1N-PN1 (G × T; *p* ≤ 0.01). This was associated with increased yield, specifically in SMARC1N-PN1, by 8% (G × T; *p* ≤ 0.05). A trial was performed to determine if the differences observed under controlled conditions would be replicated in the field. The large difference in yield between genotypes indicates that SMARC1N-PN1 is not well adapted to agronomic conditions in Manitoba. Both genotypes exhibited a limited yield response to sulfate fertilization, by 3–15% (**Table 3**). This response is typical for common bean and other legume crops.

**Table 2:** Effect of sulfur on number of seeds, seed weight, and yield under controlled conditions.

Genotype	Treatment	Number of seeds	Seed weight (mg)	Yield (g)
SARC1	LS	134 ± 16	214.6 ± 10.1	28.6 ± 2.1
	HS	143 ± 9	196.9 ± 8.9	28.2 ± 1.5
SMARC1N-PN1	LS	152 ± 12	195.5 ± 9.0	29.6 ± 1.2
	HS	157 ± 9	203.8 ± 11.8	32.0 ± 0.7

**Source of variation**	**d. f.**	**ANOVA *p*-value**		

Genotype (G)	1	0.008	n. s.	0.002
Treatment (T)	1	n. s.	n. s.	n. s.
G × T	1	n. s.	0.01	0.05
Error	15			

*Values are the average ± SD; *n* = 5; LS, low sulfur (0.2 mM SO_4_); HS, high sulfur (2 mM SO_4_); d. f., degrees of freedom; n. s., not significant*.

**Table 3:** Seed yield in sulfur deficient and -sufficient field conditions.

Genotype	Yield without sulfur (kg ha^-1^)	Yield with sulfur (kg ha^-1^)
SARC1	1736 ± 407	1995 ± 564
SMARC1N-PN1	667 ± 330	689 ± 211

*Values are the average ± SD; *n* = 4*.

### INCREASED SEED CONCENTRATION OF SULFUR AND SULFATE IN RESPONSE TO HIGH SULFATE TREATMENT

To determine if the sulfur treatment effectively altered seed composition and particularly the concentration of sulfur and its metabolites, mature seeds were analyzed for total carbon, nitrogen, and sulfur by elemental analysis and for sulfate concentration by ion analysis. Previously, SARC1 and SMARC1N-PN1 were shown to have similar nitrogen concentration in seed (Hartweck and Osborn, 1997), and Taylor et al. (2008) reported a similar seed concentration of carbon, nitrogen, and sulfur. The sulfur treatment did not change carbon and nitrogen concentration, but had a significant effect on sulfur and sulfate concentration (**Table 4**). Sulfur concentration was raised by the HS treatment by approximately 15 to 20% in both genotypes. Sulfate concentration was increased by 17% in SARC1 and 38% in SMARC1N-PN1. The differences in sulfur and sulfate concentrations indicate that treatment conditions are suitable to investigate whether the two genotypes respond differently to sulfur nutrition.

**Table 4:** Elemental and sulfate concentrations in mature seed.

Genotype	Treatment	C (%)	N (%)	S (%)	SO_4_^2-^ (nmol/mg)
SARC1	LS	46.4 ± 0.2	3.90 ± 0.11	0.20 ± 0.12	0.18 ± 0.03
	HS	46.4 ± 0.1	3.91 ± 0.17	0.23 ± 0.01	0.21 ± 0.03
SMARC1N-PN1	LS	46.0 ± 0.1	3.63 ± 0.20	0.19 ± 0.01	0.21 ± 0.05
	HS	46.0 ± 0.1	3.71 ± 0.14	0.23 ± 0.01	0.29 ± 0.02

**Source of variation**	**d. f.**	**ANOVA *p*-value**			

Genotype (G)	1	0.0001	0.006	n. s.	0.002
Treatment (T)	1	n. s.	n. s.	0.0001	0.001
G × T	1	n. s.	n. s.	n. s.	n. s.
Error	15				

*Values are the average ± SD; *n* = 5*.

### INCREASED CONCENTRATION OF CYSTEINE AND GLOBULINS IN SMARC1N-PN1 UNDER HIGH SULFATE CONDITIONS

To evaluate whether sulfur nutrition has an effect on the total concentration of sulfur amino acids, methionine, cysteine, and the non-protein amino acid, *S*-methylcysteine were quantified after acid hydrolysis of ground seed tissue. As expected, the concentration of these three amino acids was different between genotypes, methionine, and cysteine being higher, and *S*-methylcysteine lower in SMARC1N-PN1 than in SARC1 (**Table 5**), as previously reported (Taylor et al., 2008). HS increased the levels of *S*-methylcysteine by approximately 40% in both genotypes. Cysteine concentration was raised in response to the HS treatment specifically in SMARC1N-PN1, by 16% (G × T; *p* ≤ 0.03). On average, the combined levels of methionine and cysteine were elevated by 13% in SMARC1N-PN1, while they were decreased by 2% in SARC1 in response to the HS treatment.

**Table 5:** Sulfur amino acid concentration in mature seed.

Genotype	Treatment	Methionine (nmol per mg)	Cysteine (nmol per mg)	*S*-Methylcysteine (nmol per mg)
SARC1	LS	16.7 ± 0.2	22.3 ± 3.0	18.2 ± 2.0
	HS	16.7 ± 1.4	21.6 ± 2.2	23.6 ± 3.8
SMARC1N-PN1	LS	17.2 ± 1.0	24.4 ± 1.1	9.1 ± 0.4
	HS	19.0 ± 1.1	28.2 ± 1.9	12.8 ± 0.9

**Source of variation**	**d. f.**	**ANOVA *p*-value**		

Genotype (G)	1	0.04	0.0004	0.0001
Treatment (T)	1	n. s.	n. s.	0.007
G × T	1	n. s.	0.03	n. s.
Error	15			

*Values are the average ± SD; *n* = 3; *n* = 5 for cysteine*.

To investigate whether the differences in sulfur nutrient allocation influenced seed storage protein composition, an important determinant of seed quality, albumins, and globulins were sequentially extracted and their concentration quantified (**Table 6**). The concentration of extractible albumins was unchanged by the treatment. However, the concentration of extractible globulins increased specifically in SMARC1N-PN1, by 24% (G × T; *p* ≤ 0.008).

**Table 6:** Concentration of extractible albumins and globulins in mature seed.

Genotype	Treatment	Albumins (%)	Globulins (%)
SARC1	LS	1.79 ± 0.25	3.01 ± 0.51
	HS	1.88 ± 0.10	2.71 ± 0.21
SMARC1N-PN1	LS	1.95 ± 0.11	3.59 ± 0.57
	HS	1.92 ± 0.24	4.47 ± 0.31

**Source of variation**	**d. f.**	**ANOVA *p*-value**	

Genotype (G)	1	n. s.	0.0001
Treatment (T)	1	n. s.	n. s.
G × T	1	n. s.	0.008
Error	15		

*Values are the average ± SD; *n* = 5*.

**FIGURE 2:**
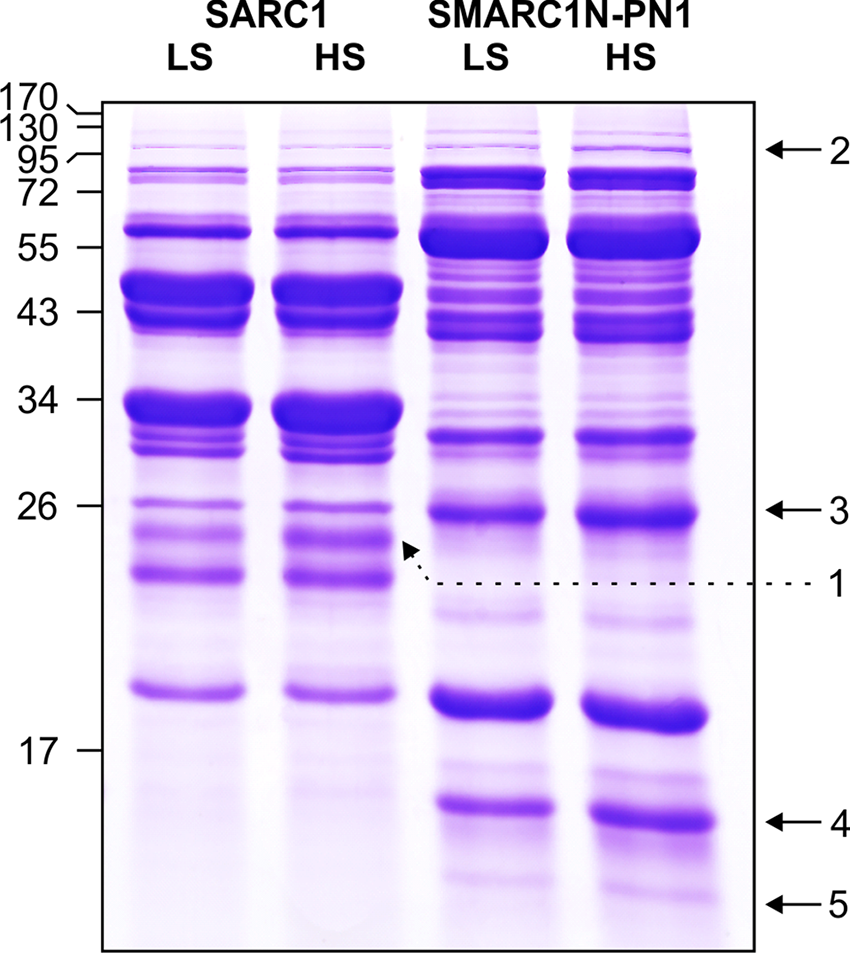
**SDS-PAGE of globulin fractions extracted from mature seeds of SARC1 and SMARC1N-PN1 grown under LS or HS.** Size of molecular mass markers is indicated on the left. Bands quantified by image analysis and excised for proteomic identification are numbered and indicated by arrows. A volume of sample equivalent to the same weight of tissue extracted was subjected to SDS-PAGE on a 12% polyacrylamide gel. The total volume of bands in each lane measured with Quantity One was as follows, SARC1-LS: 632; SARC1-HS: 636; SMARC1N-PN1-LS: 945; and SMARC1N-PN1-HS: 1007.

The globulin extracts were analyzed by SDS-PAGE. A volume of sample equal to a similar weight of seed tissue extracted was separated by electrophoresis (**Figure 2**). The volume of five protein bands appeared to be increased by the sulfur treatment. This was confirmed by image analysis with Quantity One (**Table 7**). The fold change and statistical significance of the changes was confirmed by analyzing replicate extracts of each genotype on separate gels (Supplementary Figure S1; Supplementary Tables S1 and S2). The protein bands were excised, digested with trypsin and analyzed by MALDI-MS or LC–MS–MS and identified by MASCOT search against the MASCOT database, or a translated database of the common bean genome (Schmutz et al., 2014). Protein bands no. 2 and 3 could be identified by MALDI-MS, whereas protein bands no. 1, 4, and 5 required more sensitive LC–MS–MS analysis. **Tables 8** and **9**provide information about protein identifications and list the number of methionine, cysteine, and total residues in each protein. In all cases, the apparent molecular mass measured by electrophoresis matched the predicted molecular mass relatively closely.

**Table 7:** Quantification and apparent molecular mass of sulfur-responsive protein bands identified in **Figure 2**.

Protein band no.	Apparent molecular mass (kDa)	SARC1			SMARC1N-PN1		
		Volume		Increase (%)	Volume		Increase (%)
			
		LS	HS		LS	HS	
1	25.0	29.7	44	48	–	–	–
2	105.4	6.30	6.54	4	12.4	24.9	101
3	26.5	24.5	29.1	19	142	189	33
4	15.7	–	–	–	83.2	110	32
5	13.8	–	–	–	9.64	13.1	36

**Table 8:** Identification of protein bands in **Figure 2** by MALDI-MS and MASCOT search following trypsin digestion.

Protein band no.	Sample	Accession	Predicted mass (kDa)	Protein score	*p*-value	Matches	Protein coverage (%)	Residues vs. Met vs. Cys
2	SMARC1N-PN1-LS	Lox-3	97527	68	0.03	46	33	860/15/4
2	SMARC1N-PN1-HS	Lox-3	97527	85	0.0006	27	25	860/15/4
3	SMARC1N-PN1-LS	Alb-2	25574	83	0.0009	21	73	227/2/3
3	SMARC1N-PN1-HS	Alb-2	25574	82	0.001	19	70	227/2/3

*Lox-3, lipoxygenase-3; Alb-2, albumin-2. See section “Materials and Methods” for accession numbers*.

**Table 9:** Identification of protein bands in **Figure 2** by MS-MS and MASCOT search following trypsin digestion.

Band no.	Sample	Accession	Predicted mass (kDa)	Total ions score	Matches	Sequences	Protein coverage (%)	Residues vs. Met vs. Cys
1	SARC1-LS	ARL4	26585	56	1	1	4	240/1/2
1	SARC1-HS	ARL4	26585	241	4	4	20	240/1/2
4	SMARC1N-PN1-LS	α-AI β subunit	15395	628	46	8	66	137/0/0
4	SMARC1N-PN1-LS	α-AI β subunit	15395	645	42	8	66	137/0/0
5	SMARC1N-PN1-LS	α-AI β subunit	15395	373	32	5	47	137/0/0
5	SMARC1N-PN1-HS	α-AI β subunit	15395	484	21	6	56	137/0/0

*ARL4, Arcelin-like protein 4; α-AI, α-amylase inhibitor. The α-amylase inhibitor polypeptide precursor has a total of 246 residues with four methionines and no cysteine*.

Protein band no. 2 was identified as a lipoxygenase, which was named lipoxygenase-3, based on its similarity to the corresponding soybean and pea proteins. Its baseline levels were higher in SMARC1N-PN1 than in SARC1 by approximately twofold. These results are consistent with the prior identification of this lipoxygenase as elevated in SMARC1N-PN1 as compared with SARC1, both by spectral counting (1.6-fold) and immunoblotting (2.5-fold; Marsolais et al., 2010). This protein had been identified on the basis of the soybean lipoxygenase-3 sequence (Yenofsky et al., 1988). The common bean accession shares 88% identity with soybean lipoxygenase-3, and 84% identity with pea lipoxygenase-3 (Ealing and Casey, 1988). Lipoxygenase-3 levels were increased by the sulfur treatment by approximately twofold. This was observed exclusively in SMARC1N-PN1. The apparent molecular mass determined for lipoxygenase-3 is consistent with the fact that the pea lipoxygenase A1 polypeptide, whose N-terminal sequence matched the deduced amino acid sequence of the lipoxygenase-3 cDNA, had an apparent molecular mass greater than 97.4 kDa (Domoney et al., 1990). Lipoxygenase-3 is particularly rich in sulfur amino acids with 1.7% of its residues as methionine and 0.5% as cysteine.

Protein band no. 3 was identified as albumin-2. Its baseline levels were sixfold higher in SMARC1N-PN1 than in SARC1. These results are consistent with the prior identification of albumin-2 as being elevated by about 10-fold in SMARC1N-PN1 as compared with SARC1 according to two-dimensional electrophoresis based proteomics (Marsolais et al., 2010). This protein had been identified by *de novo* sequencing, on the basis of its similarity with pea albumin-2 (Higgins et al., 1987) and mung bean seed albumin [Uniprot:Q43680], and its full-length cDNA had been subsequently cloned (Yin et al., 2011). Its transcript levels were elevated in developing seeds of SMARC1N-PN1 as compared with SARC1 (Liao et al., 2012). Albumin-2 levels were increased by the HS treatment in both genotypes, by approximately 20–30%. The sequence of albumin-2 is relatively rich in sulfur amino acids, with 0.9% of its residues as methionine and 1.3% as cysteine.

Protein band no. 1 was identified as arcelin-like protein 4 (Lioi et al., 2003). It was only present in the SARC1 extracts. This is consistent with previous spectral counting and two-dimensional electrophoresis based proteomic data (Marsolais et al., 2010). Arcelin-like protein 4 contains 0.4% its residues as methionine and 0.8% as cysteine. Protein bands no. 4 and 5 were only observed in SMARC1N-PN1. They were identified as α-amylase inhibitor β subunit (Kasahara et al., 1996). The levels of this protein had been shown to be elevated by 20-fold in SMARC1N-PN1 as compared with SARC1 by spectral counting, and this had been validated by two-dimensional electrophoresis based proteomics (Marsolais et al., 2010). The apparent molecular mass measured for protein band no. 4 is in agreement with the results obtained with α-amylase inhibitor purified from Great Northern beans (Furuichi et al., 1993). A protein band of 15.5 kDa had been identified as the β subunit by N-terminal sequencing and appeared not to be glycosylated. Protein band no. 5 is likely be a minor form of the α-amylase inhibitor β subunit lacking one or more residues at the C-terminal. Indeed, a peptide containing the sequence of the N-terminus was detected for this band. In the work by Furuichi et al. (1993) a protein band of 13.5 kDa band was tentatively assigned as the α subunit and appeared to be glycosylated. Here, the proteomic data were unambiguous and did not identify a match to the α subunit. Interestingly, the α subunit was conspicuously absent from the SMARC1N-PN1 proteome determined by spectral counting (Marsolais et al., 2010). Purification of the β subunit yielded a complex with the α-amylase inhibitor like protein, with a predicted molecular mass of 25 kDa, in which the α subunit was absent (Yin and Marsolais, unpublished results). The apparent molecular mass of this complex measured by size exclusion chromatography was equal to 41.2 kDa. In SDS-PAGE, the purified fraction contained a minor band of approximately 13.8 kDa, similar to the results in **Figure 2**. The α-amylase inhibitor β chain is devoid of sulfur amino acids. However, the corresponding α-amylase-1 precursor contains 1.6% of its residues as methionine without any cysteine (Prescott et al., 2005). The four methionines are located at the N-terminus of the polypeptide precursor. Sulfur amino acid residues are neither present in the α subunit (Kasahara et al., 1996).

## DISCUSSION

The most important finding reported from this study is that the protein pool of SMARC1N-PN1 was able to accommodate an increase in sulfur amino acids, particularly cysteine, in response to enhanced sulfate nutrition, whereas this was not the case in SARC1. This property is associated with the presence of an endogenous sink for sulfur in SMARC1N-PN1. The increase in cysteine concentration was associated with a specific increase in the concentration of extractible globulins and seed yield in SMARC1N-PN1. It has been reported that sulfur nutrition influences the response of chickpea plants to the transgenic expression of sunflower seed albumin (Chiaiese et al., 2004). An increase in the seed concentration of reduced sulfur was more pronounced in the transgenic line than in wild-type under conditions of low nitrogen and high sulfur nutrition. Further results were suggestive of a higher methionine concentration in the transgenic line in response to high sulfur and high nitrogen, although this could not be analyzed statistically due to a lack of replication. Recently, Kim et al. (2014) reported that adequate sulfate nutrition is required to maximize the accumulation of maize δ-zein in transgenic soybean lines where expression of the endogenous β-conglycinin was suppressed by RNA interference.

The seed concentration of sulfate reported here for common bean is much lower than the concentration of oxidized sulfur in lupin or pea, by approximately 100-fold (Tabe and Droux, 2002; Chiaiese et al., 2004). Whereas in lupin or pea, sulfate represents a reserve of sulfur, whose levels can be significantly reduced upon transgenic expression of a sulfur-rich protein, *S*-methylcysteine plays a similar role in common bean. This is supported by the fact that the *S*-methylcysteine concentration was reduced by approximately twofold in SMARC1N-PN1 as compared with SARC1. However, the effect of sulfur nutrition on the concentration of *S*-methylcysteine was similar between genotypes. Likewise in chickpea, nitrogen and sulfur treatments had a similar effect on oxidized sulfur concentration in wild-type and a transgenic line expressing sunflower seed albumin (Chiaiese et al., 2004).

The increase in the extractible globulin fraction associated with enhanced levels of cysteine stimulated an analysis of sulfur-responsive proteins in the extracts. Two lectins, uniquely present in either genotype, arcelin-like protein 4, and the β subunit of the α-amylase inhibitor, were identified as sulfur-responsive. In the case of the α-amylase inhibitor, sulfur is needed for the accumulation of the polypeptide precursor but not the mature β subunit. Albumin-2 was found to be increased by a similar fold change in response to HS in both genotypes, although its baseline levels were higher in SMARC1N-PN1. In pea, albumin-2 levels were initially found to be reduced in response to severe sulfur deficiency (Randall et al., 1979). In later work, protein levels were found to be relatively unchanged in response to moderate sulfur deficiency (Higgins et al., 1987). Albumin-2 is characterized by the presence of four repeats of the hemopexin domain. The results of crystallization studies have revealed that these proteins bind spermine (Gaur et al., 2010, 2011). Binding of heme and spermine was found to be mutually exclusive in grasspea hemopexin. A pea mutant lacking albumin-2 had altered levels of polyamines, and this was associated with increased seed protein concentration (Vigeolas et al., 2008). Whether SARC1 and SMARC1N-PN1 differ in their level of polyamines could be the subject of future investigation.

The identification of lipoxygenase-3 as a sulfur-responsive protein is particularly interesting, because this response was observed in only one of the genotypes. The present result strongly suggests that the sulfur-responsive albumin protein of 95 kDa identified in pea is actually lipoxygenase-3 (Higgins et al., 1987). We speculate that differences in sulfur-responsiveness between the two common bean genotypes are probably determined by polymorphisms in the promoter or 3′-untranslated region of the lipoxygenase-3 gene. These polymorphisms must arise from recombination at the lipoxygenase-3 locus between the different parents. If this is true, sequence comparisons between the two genotypes might lead to the identification of a much sought after cis-acting regulatory element determining a positive response to sulfur nutrition in higher plants. To date, the only known cis-acting regulatory motif determining a transcriptional response to sulfur, the SURE motif, is involved in the up-regulation of sulfate transporter and assimilatory genes in response to sulfur deficiency (Maruyama-Nakashita et al., 2005).

The present results have special implications for the agronomic management of common bean, if storage protein deficiency is used as a trait for the improvement of protein quality through conventional breeding. Although SARC1 and SMARC1N-PN1 responded equally to sulfate fertilization under field conditions, the results obtained under controlled conditions suggest that adequate sulfur nutrition is required to maximize the concentration of sulfur amino acids and therefore protein quality in genotypes lacking phaseolin and major lectins like SMARC1N-PN1. As deposition of sulfur due to atmospheric pollution decreases, sulfate fertilization might become necessary for common bean production in Southwestern Ontario. It is currently an integral part of agronomic production in the plains of Manitoba.

## Conflict of Interest Statement

The authors declare that the research was conducted in the absence of any commercial or financial relationships that could be construed as a potential conflict of interest.

## References

[B1] Amir R. Han T. Ma F. (2012). Bioengineering approaches to improve the nutritional values of seeds by increasing their methionine content. *Mol. Breed.* 29 915–924 10.1007/s11032-011-9690-7

[B2] Anderson J. W. Fitzgerald M. A. (2001). Physiological and metabolic origin of sulphur for the synthesis of seed storage proteins. *J. Plant Physiol.* 158 447–456 10.1078/0176-1617-00356

[B3] Arag ao F. J. L. Barros L. M. G. De Sousa M. V. Grossi De Sá M. F. Almeida E. R. P. Gander E. S. (1999). Expression of a methionine-rich storage albumin from the Brazil nut (*Bertholletia excelsa* HBK Lecythidaceae) in transgenic bean plants (*Phaseolus vulgaris* L. *Fabaceae). Genet. Mol. Biol.* 22 445–449 10.1590/S1415-47571999000300026

[B4] Blagrove R. J. Gillesie J. M. Randall P. J. (1976). Effect of sulphur supply on the seed globulin composition of *Lupinus angustifolius*. *Aust. J. Plant Physiol.* 3 173–184 10.1071/PP9760173

[B5] Bourgis F. Roje S. Nuccio M. L. Fisher D. B. Tarczynski M. C. Li C. J. (1999). S-Methylmethionine plays a major role in phloem sulfur transport and is synthesized by a novel type of methyltransferase. *Plant Cell* 11 1485–1497 10.1105/tpc.11.8.1485 10449582 PMC144290

[B6] Buchner P. Stuiver C. E. Westerman S. Wirtz M. Hell R. Hawkesford M. J. (2004). Regulation of sulfate uptake and expression of sulfate transporter genes in *Brassica oleracea* as affected by atmospheric H2S and pedospheric sulfate nutrition. *Plant Physiol.* 136 3396–3408 10.1104/pp.104.046441 15377780 PMC523398

[B7] Chandler P. M. Higgins T. J. Randall P. J. Spencer D. (1983). Regulation of legumin levels in developing pea seeds under conditions of sulfur deficiency: rates of legumin synthesis and levels of legumin mRNA. *Plant Physiol.* 71 47–54 10.1104/pp.71.1.47 16662796 PMC1065983

[B8] Chandler P. M. Spencer D. Randall P. J. Higgins T. J. (1984). Influence of sulfur nutrition on developmental patterns of some major pea seed proteins and their mRNAs. *Plant Physiol.* 75 651–657 10.1104/pp.75.3.651 16663681 PMC1066970

[B9] Chiaiese P. Ohkama-Ohtsu N. Molvig L. Godfree R. Dove H. Hocart C. (2004). Sulphur and nitrogen nutrition influence the response of chickpea seeds to an added, transgenic sink for organic sulphur. *J. Exp. Bot.* 55 1889–1901 10.1093/jxb/erh198 15234997

[B10] De Kok L. J. Stulen I. Hawkesford M. J. (2011). “Sulfur nutrition in crop plants,” in *The Molecular and Physiological Basis of Nutrient Use Efficiency in Crops*, eds Hawkesford M. J. Barraclough P. (Chichester: Wiley-Blackwell), 295–309 10.1002/9780470960707.ch14

[B11] Demidov D. Horstmann C. Meixner M. Pickardt T. Saalbach I. Galili G. Müntz K. (2003). Additive effects of the feed-back insensitive bacterial aspartate kinase and the Brazil nut 2S albumin on the methionine content of transgenic narbon bean (*Vicia narbonensis* L.). *Mol. Breed.* 11 187–201 10.1023/A:1022814506153

[B12] Dinkins R. D. Reddy M. S. S. Meurer C. A. Yan B. Trick H. Thibaud-Nissen F. (2001). Increased sulfur amino acids in soybean plants over expressing the maize 15 kDa zein protein. *In vitro Cell.* *Dev. Biol. Plant* 37 742–747 10.1007/s11627-001-0123-x

[B13] Domoney C. Firmin J. L. Sidebottom C. Ealing P. M. Slabas A. Casey R. (1990). Lipoxygenase heterogeneity in *Pisum sativum*. *Planta* 181 35–43 10.1007/BF00202322 24196672

[B14] Ealing P. M. Casey R. (1988). The complete amino acid sequence of a pea (*Pisum sativum*) seed lipoxygenase predicted from a near full-length cDNA. *Biochem. J.* 253 915–918. 3140791 10.1042/bj2530915 PMC1149390

[B15] Furuichi Y. Takemura M. Uesaka N. Kamemura K. Shimada S. Komada H. (1993). Some characteristics of an α-amylase inhibitor from *Phaseolus vulgaris* (cultivar Great Northern) seeds. *Biosci. Biotechnol. Biochem.* 57 147–148 10.1271/bbb.57.147 7763419

[B16] Galili G. Amir R. (2013). Fortifying plants with the essential amino acids lysine and methionine to improve nutritional quality. *Plant Biotechnol. J.* 11 211–222 10.1111/pbi.12025 23279001

[B17] Gaur V. Chanana V. Jain A. Salunke D. M. (2011). The structure of a haemopexin-fold protein from cow pea (*Vigna unguiculata*) suggests functional diversity of haemopexins in plants. *Acta Crystallogr. Sect. F. Struct. Biol. Cryst. Commun.* 67 193–200 10.1107/s1744309110051250 PMC3034607 21301085

[B18] Gaur V. Qureshi I. A. Singh A. Chanana V. Salunke D. M. (2010). Crystal structure and functional insights of hemopexin fold protein from grass pea. *Plant Physiol.* 152 1842–1850 10.1104/pp.109.150680 20147493 PMC2850029

[B19] Gayler K. R. Sykes G. E. (1985). Effects of nutritional stress on the storage proteins of soybeans. *Plant Physiol.* 78 582–585 10.1104/pp.78.3.582 16664286 PMC1064779

[B20] Hanafy M. S. Rahman S. M. Nakamoto Y. Fujiwara T. Naito S. Wakasa K. (2013a). Differential response of methionine metabolism in two grain legumes, soybean and azuki bean, expressing a mutated form of *Arabidopsis* cystathionine γ-synthase. *J. Plant Physiol.* 170 338–345 10.1016/j.jplph.2012.10.018 23286999

[B21] Hanafy M. S. Rahman S. M. Nakamoto Y. Fujiwara T. Naito S. Wakasa K. (2013b). Erratum to differential response of methionine metabolism in two grain legumes, soybean, and azuki bean, expressing a mutated form of *Arabidopsis* cystathionine γ-synthase. *J. Plant Physiol.* 170 1469 10.1016/j.jplph.2013.06.003 23286999

[B22] Hartweck L. M. Osborn T. C. (1997). Altering protein composition by genetically removing phaseolin from common bean seeds containing arcelin or phytohemagglutinin. *Theor. Appl. Genet.* 95 1012–1017 10.1007/s001220050655

[B23] Hawkesford M. J. (2000). Plant responses to sulphur deficiency and the genetic manipulation of sulphate transporters to improve S-utilization efficiency. *J. Exp. Bot.* 51 131–138 10.1093/jexbot/51.342.131 10938804

[B24] Hawkesford M. J. (2003). Transporter gene families in plants: the sulphate transporter gene family - redundancy or specialization? *Physiol.* *Plant.* 117 155–163 10.1034/j.1399-3054.2003.00034.x

[B25] Hawkesford M. J. De Kok L. J. (2006). Managing sulphur metabolism in plants. *Plant Cell Environ.* 29 382–395 10.1111/j.1365-3040.2005.01470.x 17080593

[B26] Herman E. M. (2014). Soybean seed proteome rebalancing. *Front. Plant Sci.* 5:437 10.3389/fpls.2014.00437 PMC4153022 25232359

[B27] Hernández-Sebastià C. Marsolais F. Saravitz C. Israel D. Dewey R. E. Huber S. C. (2005). Free amino acid profiles suggest a possible role for asparagine in the control of storage-product accumulation in developing seeds of low- and high-protein soybean lines. *J. Exp. Bot.* 56 1951–1963 10.1093/jxb/eri191 15911557

[B28] Herschbach C. Van Der Zalm E. Schneider A. Jouanin L. De Kok L. J. Rennenberg H. (2000). Regulation of sulfur nutrition in wild-type and transgenic poplar over-expressing γ-glutamylcysteine synthetase in the cytosol as affected by atmospheric H2S. *Plant Physiol.* 124 461–473 10.1104/pp.124.1.461 10982459 PMC59159

[B29] Higgins T. J. V. Beach L. R. Spencer D. Chandler P. M. Randall P. J. Blagrove R. J. (1987). cDNA and protein sequence of a major pea seed albumin (PA 2: Mr≈26 000). *Plant Mol. Biol.* 8 37–45 10.1007/BF00016432 24302522

[B30] Holowach L. P. Madison J. T. Thompson J. F. (1986). Studies on the mechanism of regulation of the mRNA level for a soybean storage protein subunit by exogenous L-methionine. *Plant Physiol.* 80 561–567 10.1104/pp.80.2.561 16664662 PMC1075155

[B31] Holowach L. P. Thompson J. F. Madison J. T. (1984). Effects of exogenous methionine on storage protein composition of soybean cotyledons cultured in vitro. *Plant Physiol.* 74 576–583 10.1104/pp.74.3.576 16663463 PMC1066728

[B32] Kasahara K. Hayashi K. Arakawa T. Philo J. S. Hara S. Yamaguchi H. (1996). Complete sequence, subunit structure, and complexes with pancreatic α-amylase of an α-amylase inhibitor from *Phaseolus vulgaris* white kidney beans. *J. Biochem. (Tokyo)* 120 177–183 10.1093/oxfordjournals.jbchem.a021381 8864861

[B33] Kelly J. D. Hefle S. L. (2000). 2S Methionine-rich protein (SSA) from sunflower seed is an IgE-binding protein. *Allergy* 55 556–560 10.1034/j.1398-9995.2000.00498.x 10858987

[B34] Kim H. Hirai M. Y. Hayashi H. Chino M. Naito S. Fujiwara T. (1999). Role of O-acetyl-L-serine in the coordinated regulation of the expression of a soybean seed storage-protein gene by sulfur and nitrogen nutrition. *Planta* 209 282–289 10.1007/s004250050634 10502094

[B35] Kim W. S. Chronis D. Juergens M. Schroeder A. C. Hyun S. W. Jez J. M. (2012). Transgenic soybean plants overexpressing O-acetylserine sulfhydrylase accumulate enhanced levels of cysteine and Bowman-Birk protease inhibitor in seeds. *Planta* 235 13–23 10.1007/s00425-011-1487-8 21805150

[B36] Kim W. Jez J. M. Krishnan H. B. (2014). Effects of proteome rebalancing and sulfur nutrition on the accumulation of methionine rich δ-zein in transgenic soybeans. *Front. Plant Sci.* 5:633 10.3389/fpls.2014.00633 PMC4227475 25426134

[B37] Kim W. S. Krishnan H. B. (2004). Expression of an 11 kDa methionine-rich delta-zein in transgenic soybean results in the formation of two types of novel protein bodies in transitional cells situated between the vascular tissue and storage parenchyma cells. *Plant Biotechnol. J.* 2 199–210 10.1111/j.1467-7652.2004.00063.x 17147611

[B38] Krishnan H. B. (2005). Engineering soybean for enhanced sulfur amino acid content. *Crop Sci.* 45 454–461 10.2135/cropsci2005.0454

[B39] Krishnan H. B. Bennett J. O. Kim W. Krishnan H. A. Mawhinney T. P. (2005). Nitrogen lowers the sulfur amino acid content of soybean (*Glycine max* [L.] Merr.) by regulating the accumulation of Bowman-Birk protease inhibitor. *J. Agric. Food Chem.* 53 6347–6354 10.1021/jf050510i 16076117

[B40] Krishnan H. B. Kerley M. S. Allee G. L. Jang S. Kim W. S. Fu C. J. (2010). Maize 27 kDa gamma-zein is a potential allergen for early weaned pigs. *J. Agric. Food Chem.* 58 7323–7328 10.1021/jf100927u 20491474

[B41] Lee M. S. Huang T. F. Toro-Ramos T. Fraga M. Last R. L. Jander G. (2008). Reduced activity of *Arabidopsis thaliana* HMT2, a methionine biosynthetic enzyme, increases seed methionine content. *Plant J.* 54 310–320 10.1111/j.1365-313X.2008.03419.x 18208517

[B42] Li Z. Meyer S. Essig J. S. Liu Y. Schapaugh M. A. Muthukrishnan S. (2005). High-level expression of maize gamma-zein protein in transgenic soybean (*Glycine max*). *Mol. Breed.* 16 11–20 10.1007/s11032-004-7658-6

[B43] Liao D. Pajak A. Karcz S. R. Chapman B. P. Sharpe A. G. Austin R. S. (2012). Transcripts of sulphur metabolic genes are co-ordinately regulated in developing seeds of common bean lacking phaseolin and major lectins. *J. Exp. Bot.* 63 6283–6295 10.1093/jxb/ers280 23066144 PMC3481216

[B44] Lioi L. Sparvoli F. Galasso I. Lanave C. Bollini R. (2003). Lectin-related resistance factors against bruchids evolved through a number of duplication events. *Theor. Appl. Genet.* 107 814–822 10.1007/s00122-003-1343-8 12819911

[B45] Malavolta E. Vitti G. C. Rosolem C. A. Fageria N. K. Guimar aes P. T. G. (1987). Sulfur responses of Brazilian crops. *J. Plant Nutr.* 10 2153–2158 10.1080/01904168709363766

[B46] Marsolais F. Pajak A. Yin F. Taylor M. Gabriel M. Merino D. M. (2010). Proteomic analysis of common bean seed with storage protein deficiency reveals up-regulation of sulfur-rich proteins and starch and raffinose metabolic enzymes, and down-regulation of the secretory pathway. *J. Proteomics* 73 1587–1600 10.1016/j.jprot.2010.03.013 20353836

[B47] Maruyama-Nakashita A. Nakamura Y. Watanabe-Takahashi A. Inoue E. Yamaya T. Takahashi H. (2005). Identification of a novel cis-acting element conferring sulfur deficiency response in *Arabidopsis* roots. *Plant J.* 42 305–314 10.1111/j.1365-313X.2005.02363.x 15842617

[B48] Molvig L. Tabe L. M. Eggum B. O. Moore A. E. Craig S. Spencer D. (1997). Enhanced methionine levels and increased nutritive value of seeds of transgenic lupins (*Lupinus angustifolius* L.) expressing a sunflower seed albumin gene. *Proc. Natl. Acad. Sci. U.S.A*. 94 8393–8398 10.1073/pnas.94.16.8393 9237987 PMC22931

[B49] Montoya C. A. Lallès J. P. Beebe S. Leterme P. (2010). Phaseolin diversity as a possible strategy to improve the nutritional value of common beans (*Phaseolus vulgaris*). *Food Res. Int.* 43 443–449 10.1016/j.foodres.2009.09.040

[B50] Morton R. L. Ellery A. J. Higgins T. J. (1998). Downstream elements from the pea albumin 1 gene confer sulfur responsiveness on a reporter gene. *Mol. Gen. Genet.* 259 309–316 10.1007/s004380050817 9749674

[B51] Naito S. Hirai M. Y. Inaba-Higano K. Nambara E. Fujiwara T. Hayashi H. (1995). Expression of soybean seed storage protein genes in transgenic plants and their response to sulfur nutritional conditions. *J. Plant Physiol.* 145 614–619 10.1016/S0176-1617(11)81272-1

[B52] Nordlee J. A. Taylor S. L. Townsend J. A. Thomas L. A. Bush R. K. (1996). Identification of a Brazil-nut allergen in transgenic soybeans. *N. Engl. J. Med.* 334 688–692 10.1056/NEJM199603143341103 8594427

[B53] Osborn T. C. Hartweck L. M. Harmsen R. H. Vogelzang R. D. Kmiecik K. A. Bliss F. A. (2003). Registration of *Phaseolus vulgaris* genetic stocks with altered seed protein compositions. *Crop Sci.* 43 1570–1571 10.2135/cropsci2003.1570

[B54] Pandurangan S. Pajak A. Molnar S. J. Cober E. R. Dhaubhadel S. Hernandez-Sebastia C. (2012). Relationship between asparagine metabolism and protein concentration in soybean seed. *J. Exp. Bot.* 63 3173–3184 10.1093/jxb/ers039 22357599 PMC3350928

[B55] Prescott V. E. Campbell P. M. Moore A. Mattes J. Rothenberg M. E. Foster P. S. (2005). Transgenic expression of bean α-amylase inhibitor in peas results in altered structure and immunogenicity. *J. Agric. Food Chem.* 53 9023–9030 10.1021/jf050594v 16277398

[B56] Randall P. J. Thomson J. A. Schroeder H. E. (1979). Cotyledonary storage proteins in *Pisum sativum*. *IV.* Effects of sulfur, phosphorus, potassium and magnesium deficiencies. *Aust. J. Plant Physiol.* 6 11–24 10.1071/PP9790011

[B57] Rerie W. G. Whitecross M. Higgins T. J. (1991). Developmental and environmental regulation of pea legumin genes in transgenic tobacco. *Mol. Gen. Genet.* 225 148–157 10.1007/BF00282653 2000086

[B58] Rolletschek H. Hosein F. Miranda M. Heim U. Götz K. P. Schlereth A. (2005). Ectopic expression of an amino acid transporter (VfAAP1) in seeds of *Vicia narbonensis* and pea increases storage proteins. *Plant Physiol.* 137 1236–1249 10.1104/pp.104.056523 15793070 PMC1088317

[B59] Sánchez E. Ruiz J. M. Romero L. (2002). Proline metabolism in response to nitrogen toxicity in fruit of French Bean plants (*Phaseolus vulgaris* L. cv Strike). *Sci. Hortic*. 93 225–233 10.1016/S0304-4238(01)00342-9

[B60] Schmutz J. Mcclean P. E. Mamidi S. Wu G. A. Cannon S. B. Grimwood J. (2014). A reference genome for common bean and genome-wide analysis of dual domestications. *Nat. Genet.* 46 707–713 10.1038/ng.3008 24908249 PMC7048698

[B61] Smith F. W. Ealing P. M. Hawkesford M. J. Clarkson D. T. (1995). Plant members of a family of sulfate transporters reveal functional subtypes. *Proc. Natl. Acad. Sci. U.S.A.* 92 9373–9377 10.1073/pnas.92.20.9373 7568135 PMC40987

[B62] Song S. Hou W. Godo I. Wu C. Yu Y. Matityahu I. (2013). Soybean seeds expressing feedback-insensitive cystathionine γ-synthase exhibit a higher content of methionine. *J. Exp. Bot.* 64 1917–1926 10.1093/jxb/ert053 23530130

[B63] Streit L. G. Beach L. R. Register J. C. Jung R. Fehr W. R. (2001). Association of the Brazil nut protein gene and Kunitz trypsin inhibitor alleles with soybean protease inhibitor activity and agronomic traits. *Crop Sci.* 41 1757–1760 10.2135/cropsci2001.1757

[B64] Tabe L. M. Droux M. (2002). Limits to sulfur accumulation in transgenic lupin seeds expressing a foreign sulfur-rich protein. *Plant Physiol.* 128 1137–1148 10.1104/pp.010935 11891268 PMC152225

[B65] Takahashi H. Kopriva S. Giordano M. Saito K. Hell R. (2011). Sulfur assimilation in photosynthetic organisms: molecular functions and regulations of transporters and assimilatory enzymes. *Annu. Rev. Plant Biol.* 62 157–184 10.1146/annurev-arplant-042110-103921 21370978

[B66] Tan Q. Zhang L. Grant J. Cooper P. Tegeder M. (2010). Increased phloem transport of S-methylmethionine positively affects sulfur and nitrogen metabolism and seed development in pea plants. *Plant Physiol.* 154 1886–1896 10.1104/pp.110.166389 20923886 PMC2996030

[B67] Taylor M. Chapman R. Beyaert R. Hernández-Sebastià C. Marsolais F. (2008). Seed storage protein deficiency improves sulfur amino acid content in common bean (*Phaseolus vulgaris* L.)*:* redirection of sulfur from γ-glutamyl-S-methyl-cysteine. *J. Agric. Food Chem*. 56 5647–5654 10.1021/jf800787y 18588315

[B68] Townsend J. A. Thomas L. A. (1994). Factors which influence the *Agrobacterium*-mediated transformation of soybean. *J. Cell Biochem.* (Suppl. 18A), 78.

[B69] Ufaz S. Galili G. (2008). Improving the content of essential amino acids in crop plants: goals and opportunities. *Plant Physiol.* 147 954–961 10.1104/pp.108.118091 18612072 PMC2442549

[B70] Vigeolas H. Chinoy C. Zuther E. Blessington B. Geigenberger P. Domoney C. (2008). Combined metabolomic and genetic approaches reveal a link between the polyamine pathway and albumin 2 in developing pea seeds. *Plant Physiol.* 146 74–82 10.1104/pp.107.111369 18024559 PMC2230549

[B71] Wu Y. Messing J. (2014). Proteome balancing of the maize seed for higher nutritional value. *Front. Plant Sci.* 5:240 10.3389/fpls.2014.00240 PMC4039071 24910639

[B72] Yenofsky R. L. Fine M. Liu C. (1988). Isolation and characterization of a soybean (*Glycine max*) lipoxygenase-3 gene. *Mol. Gen. Genet.* 211 215–222 10.1007/BF00330597

[B73] Yin F. Pajak A. Chapman R. Sharpe A. Huang S. Marsolais F. (2011). Analysis of common bean expressed sequence tags identifies sulfur metabolic pathways active in seed and sulfur-rich proteins highly expressed in the absence of phaseolin and major lectins. *BMC Genomics* 12:268 10.1186/1471-2164-12-268 PMC3115882 21615926

[B74] Zhang Y. Schernthaner J. Labbé N. Hefford M. A. Zhao J. Simmonds D. H. (2014). Improved protein quality in transgenic soybean expressing a *de novo* synthetic protein, MB-16. *Transgenic Res.* 3 1–13 10.1007/s11248-013-9777-5 24435987

[B75] Zuber H. Davidian J. C. Aubert G. Aimé D. Belghazi M. Lugan R. (2010). The seed composition of *Arabidopsis* mutants for the group 3 sulfate transporters indicates a role in sulfate translocation within developing seeds. *Plant Physiol.* 154 913–926 10.1104/pp.110.162123 20702726 PMC2949013

[B76] Zuber H. Poignavent G. Le Signor C. Aime D. Vieren E. Tadla C. (2013). Legume adaptation to sulfur deficiency revealed by comparing nutrient allocation and seed traits in *Medicago truncatula*. *Plant J.* 76 982–996 10.1111/tpj.12350 24118112

