# Supplementary material for: Differential response to sulfur nutrition of two common bean genotypes differing in storage protein composition
Source: Front Plant Sci. 2015 Feb 20;6:92. doi: 10.3389/fpls.2015.00092 (PMC4335288; doi:10.3389/fpls.2015.00092)
Supplement: Supplementary file 1 [file table_1.docx]

***Supplementary Material***

**Differential response to sulfur nutrition of two common bean genotypes differing in storage protein composition**

**Sudhakar Pandurangan^1,2^, Mark Sandercock^3^, Ronald Beyaert^2^, Kenneth L. Conn^2^, Anfu Hou^3^ and Frédéric Marsolais^1,2,*^**

^1^Department of Biology, University of Western Ontario, London, Ontario, Canada

^2^Genomics and Biotechnology, Southern Crop Protection and Food Research Centre, Agriculture and Agri-Food Canada, London, Ontario, Canada

^3^Cereal Research Centre Morden, Agriculture and Agri-Food Canada, Morden, Manitoba, Canada

*** Correspondence:** Frédéric Marsolais, Genomics and Biotechnology, Southern Crop Protection and Food Research Centre, Agriculture and Agri-Food Canada, 1391 Sandford St., London, Ontario, N5V 4T3, Canada.

Frederic.Marsolais@agr.gc.ca

**Supplementary Table 1. Quantification and apparent molecular mass of sulfur-responsive protein bands from SARC1 in Supplementary Figure 1A.**

| Protein band no. | Apparent molecular weight (kDa) | Band quantity (intensity × mm) | | | | Average | Standard deviation | Percent increase | t-test *p* value |
| --- | --- | --- | --- | --- | --- | --- | --- | --- | --- |
|  |  | R1 | R2 | R3 | R4 |  |  |  |  |
| Low Sulfur | | | | | | | | | |
| 1 | 23.2 | 12.7 | 13.6 | 16.8 | 16.3 | 14.9 | 2.0 |  |  |
| 2 | 110.3 | 7.1 | 6.7 | 5.8 | 6.1 | 6.43 | 0.6 |  |  |
| 3 | 25.4 | 17.9 | 17.3 | 17.1 | 17.5 | 17.5 | 0.4 |  |  |
| High Sulfur | | | | | | | | | |
| 1 | 23.2 | 18.0 | 20.4 | 21.7 | 24.6 | 21.2 | 2.7 | 42.3 | 0.01 |
| 2 | 110.3 | 5.99 | 7.43 | 6.20 | 7.23 | 6.71 | 0.7 | 3.7 | n. s. |
| 3 | 25.4 | 18.0 | 20.5 | 21.6 | 22.4 | 20.6 | 1.9 | 17.7 | 0.02 |
