# Supplementary material for: Differential response to sulfur nutrition of two common bean genotypes differing in storage protein composition
Source: Front Plant Sci. 2015 Feb 20;6:92. doi: 10.3389/fpls.2015.00092 (PMC4335288; doi:10.3389/fpls.2015.00092)
Supplement: Supplementary file 2 [file table_2.docx]

***Supplementary Material***

**Differential response to sulfur nutrition of two common bean genotypes differing in storage protein composition**

**Sudhakar Pandurangan^1,2^, Mark Sandercock^3^, Ronald Beyaert^2^, Kenneth L. Conn^2^, Anfu Hou^3^ and Frédéric Marsolais^1,2,*^**

^1^Department of Biology, University of Western Ontario, London, Ontario, Canada

^2^Genomics and Biotechnology, Southern Crop Protection and Food Research Centre, Agriculture and Agri-Food Canada, London, Ontario, Canada

^3^Cereal Research Centre Morden, Agriculture and Agri-Food Canada, Morden, Manitoba, Canada

*** Correspondence:** Frédéric Marsolais, Genomics and Biotechnology, Southern Crop Protection and Food Research Centre, Agriculture and Agri-Food Canada, 1391 Sandford St., London, Ontario, N5V 4T3, Canada.

Frederic.Marsolais@agr.gc.ca

**Supplementary Table 2. Quantification and apparent molecular mass of sulfur-responsive protein bands from SMARC1N-PN1 in Supplementary Figure 1B.**

| Protein band no. | Apparent molecular weight (kDa) | Band quantity (intensity × mm) | | | | Average | Standard deviation | Percent increase | t-test  *p* value |
| --- | --- | --- | --- | --- | --- | --- | --- | --- | --- |
|  |  | R1 | R2 | R3 | R4 |  |  |  |  |
| Low Sulfur | | | | | | | | | |
| 2 | 112.6 | 4.8 | 4.9 | 5.3 | 5.1 | 5.05 | 0.2 |  |  |
| 3 | 27.6 | 60.7 | 68.6 | 61.8 | 66.5 | 64.4 | 3.8 |  |  |
| 4 | 16.6 | 23.7 | 32.6 | 29.3 | 28.6 | 28.5 | 3.7 |  |  |
| 5 | 14.8 | 2.3 | 2.7 | 2.9 | 3.0 | 2.74 | 0.3 |  |  |
| High Sulfur | | | | | | | | | |
| 2 | 112.6 | 10.8 | 10.1 | 7.7 | 9.4 | 9.50 | 1.3 | 88 | 0.0005 |
| 3 | 27.6 | 92.6 | 88.0 | 77.0 | 79.6 | 84.3 | 7.3 | 31 | 0.003 |
| 4 | 16.6 | 36.1 | 38.4 | 39.0 | 34.0 | 36.9 | 2.3 | 30 | 0.008 |
| 5 | 14.8 | 4.1 | 4.2 | 4.1 | 2.8 | 3.78 | 0.6 | 38 | 0.03 |
