# Supplementary figures and images for: Differential response to sulfur nutrition of two common bean genotypes differing in storage protein composition
Source: Front Plant Sci. 2015 Feb 20;6:92. doi: 10.3389/fpls.2015.00092 (PMC4335288; doi:10.3389/fpls.2015.00092)

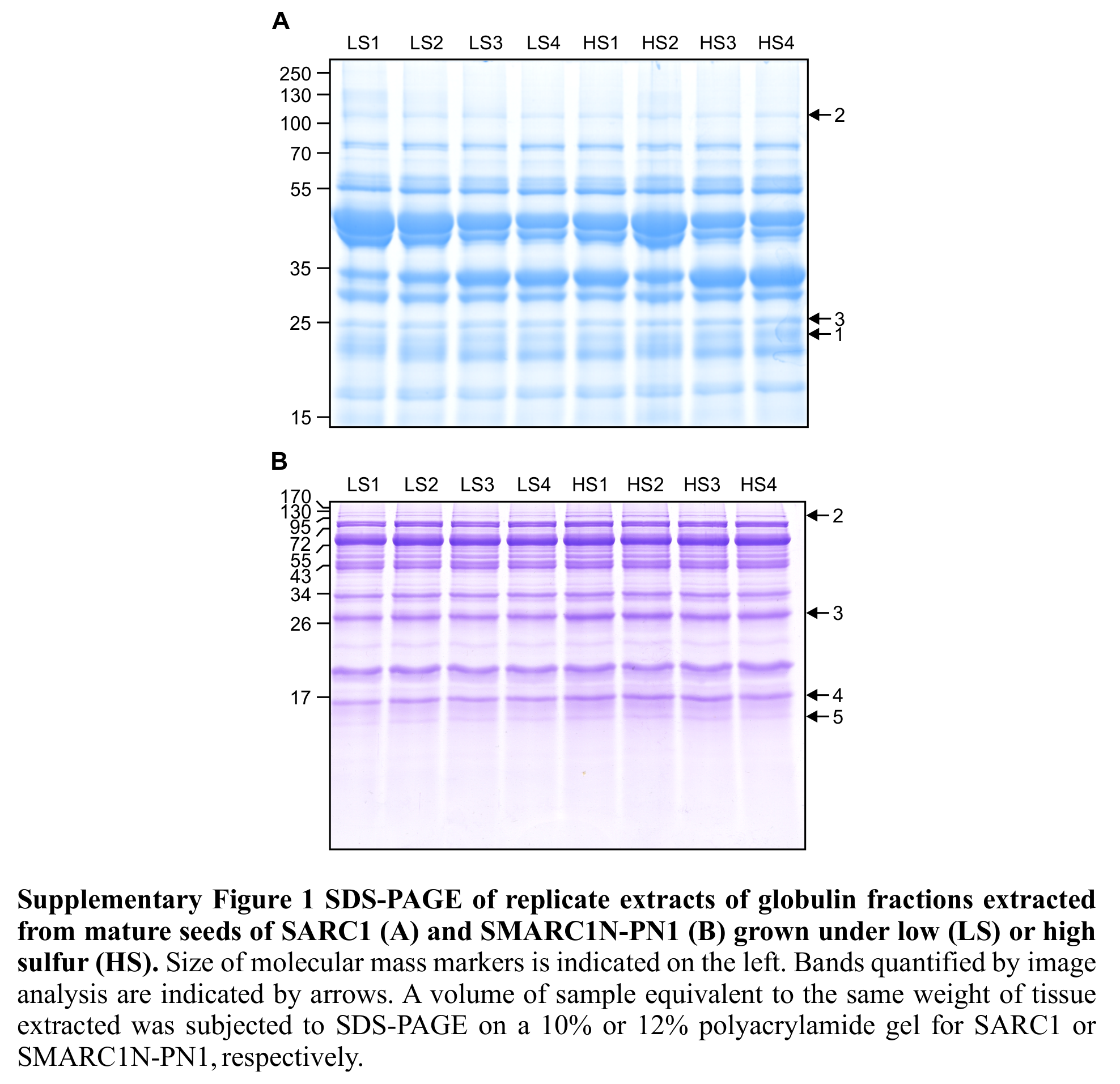

Supplement: Supplementary file 3 [file image_1.tif]
